# Optimal Cut-Off Points for Two-Step Strategy in Screening of Undiagnosed Diabetes: A Population-Based Study in China

**DOI:** 10.1371/journal.pone.0087690

**Published:** 2014-03-07

**Authors:** Zhen Ye, Liming Cong, Gangqiang Ding, Min Yu, Xinwei Zhang, Ruying Hu, Jianjun Wu, Le Fang, Hao Wang, Jie Zhang, Qingfang He, Danting Su, Ming Zhao, Lixin Wang, Weiwei Gong, Yuanyuan Xiao, Mingbin Liang, Jin Pan

**Affiliations:** 1 Zhejiang Provincial Center for Disease Control and Prevention, Hangzhou, Zhejiang, China; 2 Zhejiang University, Hangzhou, Zhejiang, China; University of Tolima, Colombia

## Abstract

To identify optimal cut-off points of fasting plasma glucose for two-step strategy in screening of undiagnosed diabetes in Chinese people, data were selected from two cross-sectional studies of Metabolic Syndrome in Zhejiang Province of China, Zhejiang Statistical Yearbook (2010), and published literatures. Two-step strategy was used among 17437 subjects sampled from population to screen undiagnosed diabetes. Effectiveness (proportion of cases identified), costs (including medical and non-medical costs), and efficiency (cost per case identified) of these different two-step screening strategies were evaluated. This study found the sensitivities of all the two-step screening strategies with further Oral Glucose Tolerance Test (OGTT) at different Fasting Plasma Glucose (FPG) cut-off points from 5.0 to 7.0 (mmol/L) ranged from 0.66 to 0.91. For the FPG point of 5.0 mmol/L, 91 percent of undiagnosed cases were identified. The total cost of detecting one undiagnosed diabetes case ranged from 547.1 to 1294.5 CNY/case, and the strategy with FPG at cut-off point of 6.1 (mmol/L) resulted in the least cost. Considering both sensitivity and cost of screening diabetes, FPG cut-off point at 5.4 mmol/L was optimized for the two-step strategy. In conclusion, different optimal cut-off points of FPG for two-step strategy in screening of undiagnosed diabetes should be used for different screening purposes.

## Introduction

Type 2 diabetes is a serious and costly disease and a growing public health problem worldwide including China, and its control and prevention has become one of major health priorities [1], [2]. Previous studies indicated that the progression of diabetes could be delayed or prevented substantially by lifestyle modification or medications [3]–[5]. Diabetes screening is an effective preventative method for catching the development of diabetes at an early stage. The increased likelihood that those with diagnosed diabetes will seek further medical advice and receive adequate treatment increases the importance of diabetes screening [6].

The most widely used screening tests include Fasting Plasma Glucose (FPG) test and Oral Glucose Tolerance Test (OGTT). FPG test is problematic as the sole screening test due to its low sensitivity, while OGTT is sufficient to diagnose diabetes mellitus. However, OGTT is not suitable for large-scaled screening, owing to its complexity and low response rate [7], [8]. So it is extremely necessary to combine the methods of FPG test and OGTT in diabetes screening or epidemiological survey. The purpose of this study was to explore the optimal PFG cut-off points for further OGTT to screen diabetes with the combination of FPG test and OGTT.

## Research Design and Methods

### Study population and sampling

A total of 19 113 individuals were selected from residents over the age of 18 years in Zhejiang province, which is located in east China and divided into 91 counties, with multistage sampling by using combination of different sampling methods: stratified random sampling in the consideration of economic status and area (city/county) to sample 15 counties in stage 1, systematic sampling to select 60 communities in stage 2 and 180 villages in stage 3, and cluster sampling in stage 4 with cluster sample of 40 families from each village.

### Measurements

Physical examination including height, weight and blood pressure, and venous blood sampling after an overnight fast of 8–14 hours, were completed for each subject after 3 days of normal carbohydrate intake and physical activity, as well as questionnaire investigation of demographic and other health related information. Plasma Glucose (PG) was measured promptly for each individual using a glucose oxidase method. For those with FPG level at the interval of [5,7) mmol/L and without diagnosed diabetes, 2 hour OGTT was performed with 75-g glucose. Diabetes mellitus was defined as FPG≥7.0 mmol/L or 2 h- OGTT PG≥11.1 mmol/L, according to the 1999 World Health Organization diagnostic criteria [9].

### Analytical procedures

#### Estimation of the proportion of undiagnosed diabetes among population with FPG less than 5.0 mmol/L

**Figure 1:**
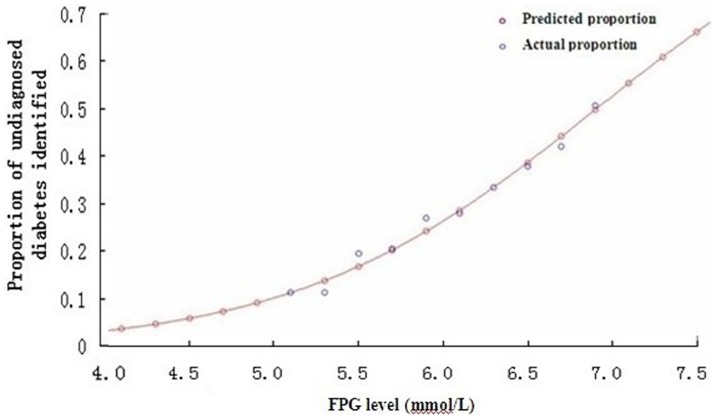
Actual proportion of undiagnosed diabetes identified at different Fasting Plasma Glucose (FPG) levels and its model prediction.

Using data available for population sample with FPG level ≥5.0 mmol/L, a model for predicting the proportion of undiagnosed diabetes by FPG level was built and it demonstrated very well (F = 303, *P*<0.001, *R* ^2^ = 0.97; see Figure 1). The mathematical model was listed as follows:
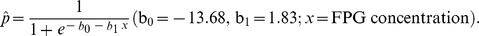
According to the above prediction model, the proportion of undiagnosed diabetes among population with FPG less than 5.0 mmol/L is estimated to be 0.56%. The authors used this model to estimate the proportion of undiagnosed diabetes for samples with FPG less than 5.0 mmol/L, and then further data analyses were conducted with these modeled data as supplement.

#### Effectiveness evaluation for each detection strategy

The effectiveness of a screening strategy was measured by the proportion of undiagnosed diabetes cases identified, which by definition is equal to the sensitivity of the screening strategy. Specificity of each screening was also presented. For the effectiveness evaluation, the combined method of FPG test and further OGTT for individuals with FPG less than 7.0 mmol/L was employed as the golden-criteria for diabetes screening.

#### Estimating the cost and efficiency of each screening strategy

Both medical and non-medical costs were included to estimate the cost of each screening strategy. Medical cost included costs for laboratory tests, personnel time, and other materials (e.g., cost of report printing). Non-medical cost was consisted of transportation fee to a health care provider and cost of patient's time spent traveling and receiving tests. The cost of identifying one diabetes case was calculated as the total cost of a screening strategy divided by the total number of cases identified. All costs were expressed in Chinese Yuan (CNY), and CNY to U.S. Dollar (USD) exchange rate was 0.1465 in 2010, the year this investigation was conducted.

Data analyses were performed with Matlab [10] and SPSS 16.0 software.

### Ethics Statement

This study was reviewed and approved by the institutional review boards of Zhejiang Provincial Center for Disease Control and Prevention, China. Each participant provided written informed consent to participate in this study.

## Results

### Characteristics of subjects

A total of 17 437 subjects, including 8 169 (46.85%) males and 9 268 (53.15%) females, were finally included in this study, with a response rate of 91.23%. The average age of this sample was 50.13±15.22. A previous of diabetes diagnosis was reported by 905 subjects of them, and 622 potential diabetes cases were identified by FPG test and OGTT.

### Effectiveness of the screening strategy

**Figure 2:**
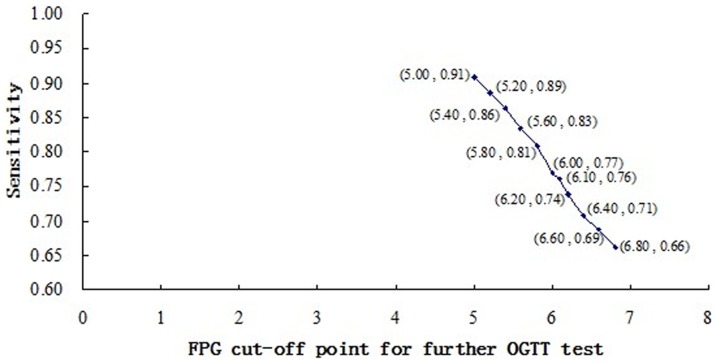
Sensitivity for each screening strategy of FPG test combined with further.

The sensitivities of all the screening strategies of FPG test combined with further OGTT test at different FPG cut-off points from 5.0 to 7.0 (mmol/L) ranged from 0.66 to 0.91 (Figure 2). The lower the FPG cut-off point was, the higher sensitivity the combined screening gained. In addition, specificities for all these combined screening strategies above were at the same value, equal to 1.00.

### Efficiency of the screening strategy

As Table 1 shown, medical, non-medical and total costs for one-time FPG test were 7.8, 8.3, 16.1 (CNY), respectively, while costs for an OGTT test were 11.8, 27.5, 39.3 (CNY). At the FPG cut-off point of 6.1 (mmol/L) for further OGTT, the total cost per case identified was the least and only 547.1 CNY was needed for early detection of one diabetes case. But the combined method of FPG test and further OGTT for all individuals with FPG less than 7.0 mmol/L was the most expensive, with a cost of 1294.5 CNY to identify an potential diabetes patient. From the perspective of marginal cost per case identified, the cut-off point of 6.8 is the best. See Table 2.

**Table 1:** Medical and non-medical costs for FPG test and OGTT test.

Cost (CNY/case)	FPG test	OGTT test
**Medical cost**	**7.8**	**11.8**
-lab test	2.8	6.8
-personnel time	4.9	4.9
-other materials	0.1	0.1
**Non-medical cost**	**8.3**	**27.5**
-patient time	5.8	25.0
-transportation	2.5	2.5
**Total cost**	**16.1**	**39.3**

**Table 2:** Comparison of cost for each screening strategy of FPG test combined with further OGTT test at different FPG cut-off points.

FPG cut-off point	Sample size		Frequency of further OGTT test	Number of undiagnosed diabetes cases identified	Cost per case identified (CNY/case)			Marginal cost per case identified (CNY/case)		
	N	%			Medical	Non-Medical	Total	Medical	Non-Medical	Total
≥7.0	436	2.7	0	436	292.7	311.5	604.2			
≥6.8	503	3.1	67	454	282.9	303.2	586.0	45.5	102.2	147.7
≥6.6	593	3.6	157	472	274.3	296.9	571.2	57.4	138.0	195.4
≥6.4	714	4.4	278	485	269.9	295.8	565.7	110.1	255.9	366.0
≥6.2	886	5.4	450	506	262.7	292.8	555.6	96.4	223.5	319.9
≥6.1^a^	1000	6.1	564	522	257.2	289.9	547.1	83.3	198.2	281.4
≥6.0	1152	7.0	716	528	257.7	294.5	552.2	301.2	694.7	995.9
≥5.8	1570	9.6	1134	554	254.5	301.4	555.9	189.5	441.5	631.0
≥5.6	2175	13.3	1739	572	259.0	321.0	580.0	397.5	924.2	1321.7
≥5.4	2998	18.3	2562	592	266.6	348.4	615.1	484.0	1132.0	1616.0
≥5.2	4044	24.7	3608	608	279.9	386.6	666.5	772.0	1800.0	2572.0
≥5.0	5001	30.6	4565	622	291.8	420.2	711.9	808.6	1879.4	2688.0
≥0	16362^b^	100.0	15926^c^	687^d^	459.3	835.2	1294.5	2062.1	4806.4	6868.6

a The cut-off point of 6.1 mmol/L was added, because it is the FPG threshold for diagnosing Impaired Fasting Glycaemia (IFG).

b Subjects with previous diabetes diagnosis, and another 170 cases with FPG in the interval [5.0, 7.0) but without further OGTT test were excluded from this analysis.

c For those cases with FPG in the interval [0, 5.0), no further OGTT test was conducted in this study.

d According to the mathematical modeling, a total of 65 undiagnosed diabetes cases were supposed to be identified by further OGTT test after FPG test among the cases.

### Combination of effectiveness and efficiency for diabetes screening

**Figure 3:**
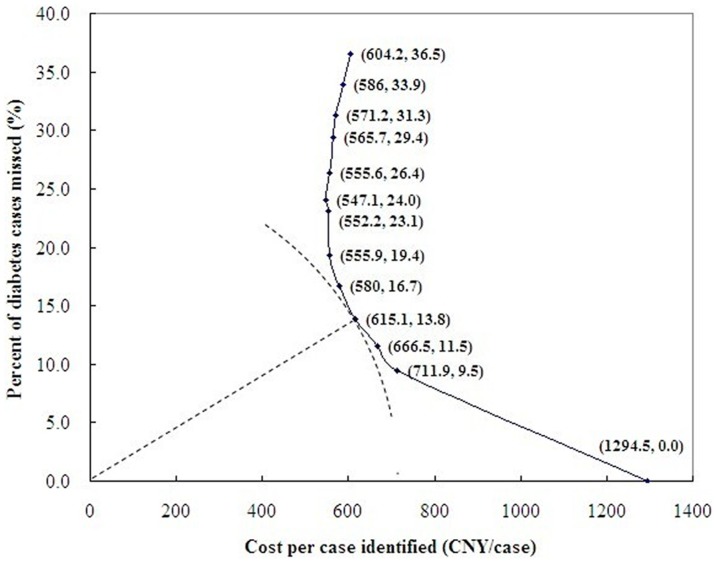
Percent of diabetes cases missed and cost per case identified by each screening strategy of FPG test combined with further OGTT test at different FPG cut-off points.

The percent of diabetes cases missed and the cost per case identified by each screening strategy of FPG test combined with further OGTT test at different FPG cut-off points are presented in Figure 3. The horizontal and vertical axes represent the cost per case identified and the percent of cases missed, respectively. The curve (known as efficiency frontier) in Figure 3 indicates the combination of effectiveness and efficiency that could be achieved by each screening strategy evaluated. The closer a point is to the origin, the better the screening strategy represented by that point.

As the Figure 3 and Table 2 shown, the point (615.1, 13.8) at the FPG cut-off of 5.4 mmol/L is the closest one. It indicates that the screening strategy represented by this point achieved the best combination of effectiveness and efficiency for diabetes screening, in other words, 5.4 mmol/L is the best cut-off point for further oral glucose tolerance test after FPG test to screen diabetes, considering both effectiveness and cost of diabetes screening.

## Conclusions

Guidelines for diabetes screening are mostly based on clinical studies and expert recommendation, so population-based study was proposed for more strong evidence. For this proposal, this current study was conducted to find the most suitable strategy for large-scale diabetes screening, using large population samples other than clinical samples or population at high risk. This is one of the strengths of this study.

The primary utility of screening is to permit the early identification of subjects who have already developed the disease, or are at imminent risk of disease development, and who can then be referred for further evaluation. Specifically, diabetes screening can serve as a tool to identify diabetes patients and people with pre-diabetes (either Impaired Glucose Tolerance [IGT] or Impaired Fasting Glucose [IFG]) for diabetes control and prevention [11], [12]. Due to limited medical resources in developing countries including China, the control of diabetic patients was list on the agenda with more attention than diabetes prevention from pre-diabetes now. Therefore, it is urgent to figure out a good strategy for screening undiagnosed diabetes.

The American Diabetes Association recommended use of FPG for screening for its significant advantages in terms of convenience and cost, lowering diagnostic thresholds from 7.8 to 7.0 mmol/L [13]. But previous studies showed that FPG alone did not have sufficient sensitivity to detect diabetes [14], [15]. Based on the criteria of FPG≥7.0 mmol/l for diabetes screening, only 63.46% (436/687) of potential diabetes patients were detected in this study, similar with other study in China [16]. If additional OGTT was used for screening, the rest 36.54% potential diabetes cases could be identified. It indicates a large proportion of diabetic subjects would have been missed if only FPG was used as the screening test for diabetes. Although OGTT was ‘platinum standard’ for diabetes diagnosis [17], [18], large scale OGTT screening was not recommended for several reasons, including inconvenience, low response rate, low reproducibility, high variability and several complicating factors (e.g. age, medication and psychological status including stress). Therefore, it is extremely necessary to combine FPG test and OGTT to take full advantage of both tests in diabetes screening.

It is commonly agreed that screening should start with non-invasive and inexpensive risk score questionnaire, and only those at moderate or high risk be included in glucose testing. For the situation in China, every person have a routinely health examination paid by government every two year or annually, so every person have gotten a test of fasting plasma glucose. Based on these health examination data, we skip the process of diabetes risk score evaluation to make full use of these data. So we only explored the combined screening strategy of FPG and OGTT in our study.

To select the most suitable strategy for screening undiagnosed diabetes, the effectiveness and efficiency of each strategy need to be considered [17], [18].The optimal FPG cut-off point for further OGTT after FPG test varies, depending on the goal of the screening program. If the screening program is to identify more diabetes cases, the screening strategy with higher sensitivity will be chosen. For this aim, the FPG cut-off point for additional OGTT is recommended to be set at 5.0 mmol/L or lower. This cut-off point was employed in this study and only 65 (9%) potential diabetes cases were missed. To detect these 65 cases, additional 11361 OGTT tests were needed. For the screening by FPG, it may miss the potential diabetes cases characterized by postprandial hyperglycemia, so the sensitivities of FPG and the two-step strategy we used may not adequate [19]. However, the screening cost should also be considered. If the program is to pursue the lowest cost per case identified, 6.1 mmol/L is suggested to be the cut-off point at lowest cost of 95.1 CNY with a relatively low sensitivity (76%). In the view of health care practice, both effectiveness and cost should be carefully considered to gain the best benefit from the screening program. In this regard, 5.4 mmol/L is the favorable FPG cut-off point for further OGTT, with adequate sensitivity (86.2%) and acceptable cost (106.8 per case). All the three above-mentioned cut-off points could be applied to meet the needs of different screening programs.

Study limitations merit note. Firstly, individuals with FPG level below 5.0 mmol/L had not been given a further OGTT test, the effectiveness and efficiency of each screening strategy was evaluated with part data from mathematical modeling, so this evaluation may not exactly reflect the truth. Secondly, the cost of personnel time and other non-medical cost were not included in the cost analysis for each screening strategy. In addition, more screening methods such as Hemoglobin A1c (HbA1c) are needed to be combined and further explored.
